# Single-molecule tracking of myelin basic protein during oligodendrocyte differentiation

**DOI:** 10.1017/S2633903X23000259

**Published:** 2023-11-20

**Authors:** Sayed M. Rassul, Masahiro Otsu, Iain B. Styles, Robert K. Neely, Daniel Fulton

**Affiliations:** 1 Neuroscience and Ophthalmology Research Group, Institute of Inflammation and Ageing, College of Medical and Dental Sciences, University of Birmingham, Birmingham, UK; 2 Physical Sciences of Imaging in the Biomedical Sciences Training Programme, University of Birmingham, Birmingham, UK; 3 Braizon Therapeutics, Inc., Kanagawa, Japan; 4 School of Electronics, Electrical Engineering and Computer Science, Queen’s University Belfast, Belfast, UK; 5 School of Chemistry, University of Birmingham, Birmingham, UK

**Keywords:** myelin, myelin basic protein, oligodendrocyte, single-molecule tracking, TIRF imaging

## Abstract

This study aimed to expand our understanding of myelin basic protein (MBP), a key component of central nervous system myelin, by developing a protocol to track and quantifying individual MBP particles during oligodendrocyte (OL) differentiation. MBP particle directionality, confinement, and diffusion were tracked by rapid TIRF and HILO imaging of Dendra2 tagged MBP in three stages of mouse oligodendroglia: OL precursors, early myelinating OLs, and mature myelinating OLs. The directionality and confinement of MBP particles increased at each stage consistent with progressive transport toward, and recruitment into, emerging myelin structures. Unexpectedly, diffusion data presented a more complex pattern with subpopulations of the most diffusive particles disappearing at the transition between the precursor and early myelinating stage, before reemerging in the membrane sheets of mature OLs. This diversity of particle behaviors, which would be undetectable by conventional ensemble-averaged methods, are consistent with a multifunctional view of MBP involving roles in myelin expansion and compaction.

## Impact Statement

Myelin basic protein (MBP) is essential for the healthy development and functioning of the central nervous system. In part, this is due to its roles in driving the wrapping and compaction of the oligodendrocyte (OL) myelin sheath. However, MBP may be involved in other functions including cytoskeleton regulation, signal complex formation, and neurite outgrowth. Considering this multifunctionality, we expect MBP particles to display a wide spectrum of behaviors depending on developmental and physiological conditions. Identifying these behaviors could provide information on MBP functions and new insight into fundamental process shaping CNS development. Workflows enabling tracking and analysis of single MBP molecules within myelin-forming OL have not previously been described. To address this gap, we developed an approach to track individual MBP particles during key stages of OL maturation and myelination. Fast-acquisition TIRF and HILO imaging of Dendra2-tagged MBP in primary cultures of developing OL allowed us to quantify various aspects of MBP motion behaviors, revealing, for the first time, distinct subpopulations of MBP particles, whose motion profiles, and temporally restricted appearance, match with current models of myelin compaction and wrapping. This approach will be useful for exploring the molecular mechanisms of MBP function in myelination and beyond.

## 1. Introduction

Myelin basic protein (MBP) is a key constituent of central nervous system myelin, representing approximately 30% of the total protein content. MBP is most prominently associated with roles in myelin wrapping and compaction^(^^1^^,^^2^^)^ as illustrated by the MBP deficient shiverer mouse strain, where a large multi-exon deletion causes loss of the major dense line, hypomyelination, generalized tremors, and a shortened lifespan.^(^^3^^–^^5^^)^ Several recent studies have advanced our understanding of the mechanisms mediating MBP’s functions during myelination. Molecular insights into its role in compaction derive from data revealing that MBP drives myelin biogenesis using phase interactions.^(^^6^^)^ These phase interactions are mediated by hydrophobic interactions from the phenylalanine residues in MBP, with the resulting structures being implicated in the formation of a structural mesh that drives compaction. By acting as a kind of molecular sieve this mesh is suggested to play a role in the extrusion of other proteins, such as CNPase and myelin-associated glycoprotein, leading to the pattern of compact and noncompact regions that characterize the mature myelin sheath.^(^^6^^,^^7^^)^ MBP is also associated with events occurring upstream of myelin compaction, with recent data supporting a model where MBP drives axonal wrapping via a mechanism involving PIP2 binding, and the disassembly of actin networks previously involved in driving axonal contact and ensheathment.^(^^8^^)^ MBP may also serve roles that extend beyond myelination.^(^^9^^,^^10^^)^ in vitro studies indicate it forms interactions with numerous molecules including cytoskeletal molecules, and proteins containing SH3 domains, implying roles in cytoskeleton regulation, signaling complex organization, and the regulation of processes such as proliferation and neurite outgrowth.^(^^11^^–^^13^^)^ Considering this multifunctionality, the population of MBP particles within a given structure is likely to display diverse behaviors, identifiable as subpopulation, that may fluctuate in abundance due to developmental and physiological influences. Studying these subpopulations could leverage new information on classic and novel MBP functions, and provide new knowledge on the fundamental process of myelination.

Previous work to observe the behavior of MBP has mainly utilized the benefits of ensemble-averaged imaging,^(^^14^^)^ which looks at the population of molecules as a whole.^(^^15^^)^ This approach has been widely adopted within the life sciences since it is easily implemented, can provide rapid observations of molecular behaviors, and due to its focus on the average behavior, helps to simplify data analysis and smooth out anomalous behaviors.^(^^16^^,^^17^^)^ However, in reality, molecules in living cells exist in subpopulations that exhibit a range of behaviors and movement over a range of different time scales.^(^^18^^)^ Due to the averaged response they detect, ensemble techniques are less sensitive to this rich variation in molecular kinetics and behaviors, thus subpopulation behaviors are overlooked. Understanding the behavior of subpopulations is important in understanding the true interactions and consequences of molecular fluctuations in a sample.^(^^19^^)^ Importantly, cultures of genetically homogenous cell populations may show identical results on an ensemble level that mask inherent variations in physical, chemical, and biological properties present at the level of molecule subpopulations.^(^^16^^)^ This intrinsic heterogeneity is valuable in the adaption of the cells to the environment, and therefore key to survival.^(^^20^^,^^21^^)^ Also, many of the conventional imaging modalities require cells to be fixed which causes distortions on the true structure of proteins.^(^^22^^)^ It is in this context that live-cell single-molecule imaging (SMI) has facilitated further understanding of cell biology.

SMI techniques allow observation of the stochastic and probabilistic behaviors which drive biological reactions, and the interplay of molecular interactions and random collisions.^(^^23^^)^ The fundamental point of SMI is the ability to identify individual molecules within the population, observe their precise location, and follow them in the cellular environment, features that are not possible with ensemble-averaged imaging.^(^^24^^)^ SMI methods are therefore ideal for investigating the behavior of MBP in different cellular structures and identifying subpopulations of MBP particles associated with distinct functions during oligodendrocyte (OL) myelination. SMI techniques can be split into two categories, single molecule localization, and single-molecule tracking (SMT).^(^^25^^,^^26^^)^ Single-molecule localization typically involves high-resolution imaging methods, such as PALM/STORM, whose greater resolving powers are achieved at the expense of temporal resolution due to their inherently slower image acquisition rates.^(^^27^^)^ SMT on the other hand aims to capture the dynamic behavior of a system by following the particle’s point spread function via fast-acquisition imaging in live samples.^(^^28^^)^ For a detailed discussion of the theoretical and practical underpinnings of SMT readers are directed to the following excellent reviews.^(^^17^^,^^29^^)^ However, in brief, SMT provides information on the motion of particles including diffusion rates and directionality,^(^^30^^)^ thus it is an ideal option for studies aiming to explore the dynamics of MBP during OL maturation. Accordingly, we developed a workflow combining rapidly transducing viral vectors encoding fluorescent photoswitchable MBP fusion proteins, with SMT from TIRF and HILO time-lapse images. SMT was performed *in vitro* using primary OL precursor cells (OPC), early myelinating OL differentiated from primary OPC cultures, and mature myelinating OL in mixed glial cultures. This protocol allowed us to characterize, for the first time, the patterns of MBP particle motion associated with OL maturation and myelination in primary OL cells, and identify novel subpopulations of MBP particles associated with these events. Collectively, this imaging protocol, and the observations it has enabled, provide a platform for exploring the molecular interactions governing MBP functionality during developmental myelination, and following CNS injury and disease events.

## 2. Materials and methods

### 2.1. Mixed glial cultures for imaging

Neonatal mice (postnatal day 0–2) were sacrificed using Home Office-approved methods (proscribed by the Animals Act, 1986). Brains were dissected out and submerged in ice-cold dissection medium, consisting of DMEM:F12 (D6421, Sigma), 1% L-glutamine (A2916801, Gibco), and 1% Pen Strep (15140122, Gibco). The cortices of the brains were separated from the midbrain and cerebellum. The meninges were removed using fine forceps, and the brains were homogenized by trituration followed by digestion in trypsin (25200056, Gibco) and DNase (EN0521, Thermo Scientific) for 20 minutes at 37°C. Cells were diluted in DMEM (41965039, Gibco) with 10% FBS (F7524, Sigma), 1% Pen Strep (15070, Thermofisher) (serum medium), and centrifuged at 300 × g for 5 minutes at 10°C. Following resuspension of the pellet cells were seeded into poly-d-lysine coated imaging dishes (P35GC-0-14-C, Mattek) at a density of 5 × 10^5^ cells. The cultures were then incubated at 37°C/8% CO_2_ for 16 hours to allow the cells to settle, followed by a complete media change. Cells were then cultured for 9–12 days, with a ⅔ media change (supplemented with 3 μg/mL insulin) performed every third day. Mixed glial cultures were used for imaging at 15 DIV.

### 2.2. OPC culture

Purified OPC cultures were isolated from mixed glial cells using the methods described above. Following centrifugation and resuspension, cell from two brains were plated into a poly-l-lysine (P9155, Sigma) coated T75 flask and cultured in serum medium at 37°C/8% CO_2._ After 24 hours flasks received a full medium change with serum medium supplemented with insulin (2 μg/mL) (I5500, Sigma), with subsequent ⅔ media changes scheduled every third day (supplemented with 3 μg/mL insulin). Once an astrocyte monolayer had formed, and the proliferation of putative OPCs (appearing as small rounded profiles) confirmed, the flasks were secured on an orbital shaker mounted inside a humidified incubator (37°C/5% CO_2_). The flasks were then shaken at 160 rpm for 30 minutes, after which the media was changed to remove microglia, and the flasks returned to the incubator and shaken for a further 16 hours at 220 rpm (37°C/5% CO_2_) to dislodge OPC from the astrocyte monolayer. OPC were then isolated from contaminating microglia based on a differential adhesion protocol. Here, the resulting cell suspension containing OPC and microglia was transferred into a noncoated 10 cm dish, and left to incubate at 37°C for 30 min with gentle swirling every 10 min to prevent adhesion of OPCs. The cell suspension containing OPC was then collected and centrifuged at 1000 rpm for 5 minutes at 10.C. The pellet was resuspended in OPC media, containing DMEM, 1% N2 (17502001, Thermofisher), 1% B27 (17504, Thermofisher), 2 nM l-Glutamine, 1% Pen Strep, 20 ng/ml rhFGF2 (PHG0266, Thermofisher), 100 ng/ml IGF1 (PHG0078, Thermofisher), 20 ng/ml PDGF-AA (PHG0035, Thermofisher), 3.6 ng/ml Hydrocortisone (H0888, Sigma), and plated in imaging dishes at a density of 5 × 10^5^ cells.

### 2.3. OPC differentiation

In order to differentiate OPC, the cells were exposed to OPC media lacking fibroblast growth factor 2 (rhFGF2), PDGF-AA, and Insulin-like growth factor 1 (IGF1), and supplemented with Triiodothyronine (T3) (T6397, Sigma) (referred to as differentiation media). Cells were then maintained with media changes every other day for 1 week, and their differentiation status examined by immunofluorescent analysis of the OPC maker protein NG2. NG2 Immunostaining, performed according to methods described by Begum, Otsu (31), revealed a robust NG2 signal in OPC cultures that was undetectable following differentiation treatment (data not shown) confirming the efficacy of this differentiation protocol.

**Figure 1.**
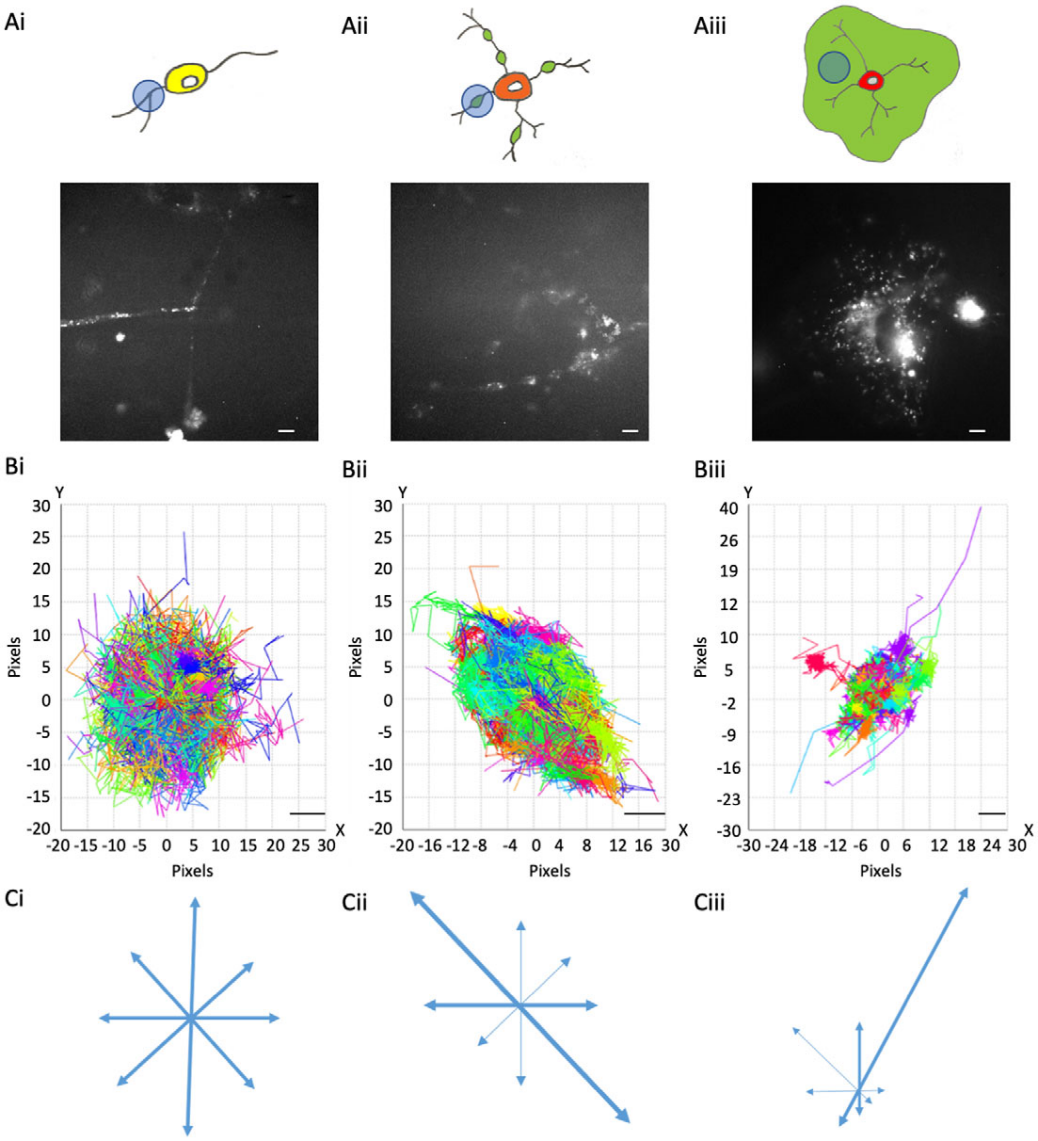
MBP::Dendra2 expression and motion profiles in oligodendroglial cells at distinct stages of maturation. (a) MBP::expression in OPC processes (a-i), eOL membrane bubbles (a-ii), and mOL sheets (a-iii). Upper panels depict representative cell morphologies with imaging regions of interest highlighted by light blue circles. Green areas in (a-ii) and (a-iii) indicate putative regions of myelination. Lower panels display representative MBP::Dendra2 signals from ROIs depicted in upper panels. Scale bars 5 μm. (b) Motion Profiles for MBP particles imaged in OPC processes (b-i), eOL membrane bubbles (b-ii), and mOL membrane sheets (b-iii). Note, all particle tracks are presented for a given imaging field to provide an overview of directionality at each developmental stage. Scale bars 1 μm. (c) Vector diagrams derived from the motion profiles shown in (b-i) (OPC), (b-ii) (eOL), and (b-iii) (mOL). Thicker arrows indicate vectors with greatest particle density.

The mature OLs observed were split into two categories, eOL and mOL. eOL were observed as multipolar cells with processes bearing small regions of flattened membrane similar to the membrane bubbles previously identified during the early stages of myelination.^(^^32^^)^ Importantly, these flattened membrane bubbles were quite distinct from the continuous areas of flattened myelin, often termed myelin sheets, often observed to surround mature myelin-forming OL *in vitro.*^(^^33^^,^^34^^)^ In this article, we refer to the flattened membrane regions produced by eOL as membrane bubbles (Figure 1). For imaging purposes, the analysis of eOLs focussed exclusively on these regions. In our hands differentiation treatment failed to convert OPC beyond the eOL stage thus it was not possible to study myelin sheets using this approach. Therefore, to study the continuous membrane sheets associated with compact myelin we imaged mOL in mixed glial cultures, where mOL were frequently observed to exhibit the “fried-egg” morphology of membrane sheets associated with fully mature myelin *in vitro* (Figure 1).

### 2.4. Viral induction

In order to visualize MBP in living cells a recombinant Semliki Forest Virus (SFV) vector of subtype A7(74) (SFVA7(74)) was used to transduce OL with a Dendra2::MBP construct. cDNA encoding Dendra2 was obtained from Dr Peter Dedecker (Ku Leven, Leuven, Belgium). Sequences for MBP were provided by Professor Mikael Simons (Institute of Neuronal Cell Biology, Technical University Munich, Munich, Germany), and cDNA for the SFVA7(74) vector were obtained from Markus Ehrengruber (Kantonsschule Hohe Promenade, Zurich). The Dendra2::MBP construct was assembled by standard PCR cloning methods and ligated into the SFVA7(74) vector. We note that our new Dendra2::MBP construct lacked the spacer region used by the Simons group in their studies of MBP diffusive mobility.^(^^6^^)^ Infectious particles were generated from the resulting SFVA7(74)-Dendra2::MBP vector in BHK cells according to methods reported by Ehrengruber and Schlesinger (35). Unpurified viral preparations were then aliquoted and stored at −80°C. SFVA7(74)-Dendra2::MBP was applied to cells when more than 20% of putative oligodendroglia possessed processes approximately 1.5 times the length of the soma on visual inspection. The cells were submerged in viral solution containing 60,000 infectious units/ml and left to incubate for 1 hour at 37°C. Following the incubation, the viral solution was washed off and the cells were incubated in media lacking phenol red for 6 hours at 37°C to allow sufficient time for transgene expression, after which time the cells were used for imaging.

### 2.5. Imaging

Regions of interest were centered on processes, membrane bubbles, and sheets since these are the key region in terms of MBP function. Imaging dishes containing the SFV-infected cells were imaged on an inverted wide-field microscope (Rapid Automated Modular Microscope, Applied Scientific Instrumentation) equipped with a 100× (1.4 NA) oil immersion TIRF objective (Nikon). The cells were first surveyed under bright-field to find cells with healthy-appearing processes, after which the microscope was converted to TIRF mode for OPC and eOL in purified cultures, and HILO mode for mOL in mixed glia cultures. Upon discovery of a process, the cells were imaged at 488 nm excitation to confirm the expression of Dendra2 in the selected process. The fluorescent cells were then primarily irradiated with 405 nm light for 5 seconds to induce Dendra2 photoswitching from a 488 nm excitation to 561 nm excitation, producing a spatially sparse red signal, which could be tracked (Figure 1). Time-lapse imaging was performed on the selected region of interest with excitation from a 561 nm laser line whose power was carefully adjusted to achieve effective excitation whilst minimizing photobleaching. Images were acquired using an Evolve Delta EM-CCD camera (Photometrics) set to 30 ms exposure times across 2500 frames. In the case of exceptionally sparse signals, or rapidly photobleaching samples, an image was taken at 488 nm to produce a reference image to compare the detected particles to, thus ensuring the resulting particle tracks were localized within a bona fide process, rather than existing as free-floating material within the dish. Following imaging the resulting time-lapse image-stacks were processed using ICY bioanalysis software.^(^^36^^)^

### 2.6. Data analysis

Image stacks were imported into ICY bioanalysis for particle detection and tracking.^(^^36^^–^^38^^)^ The images were opened and the contrast within the images adjusted to aid the detection of the processes/sheet by eye. Regions of interest were then constructed around the processes/sheet of the cell within the first image of the stack. The region of interest was verified with the other images within the stack to ensure it encompasses the process throughout the stack, and adjusted to ensure the region of interest was tightly confined to the process of the cell (Figure 1). In cases where the process moved during the imaging session, the longer segment where the process was stationary was taken forward for imaging, or the images were excluded from analysis. This region of interest was then connected to the Spot Detector function, which was set to expect detected particles with an approximate diameter of 3 pixels on a dark background, an assumption based on previous findings.^(^^39^^)^ Following particle detection, the detected particles were ported to the Particle Tracker and analyzed using the parameters shown in Table 1. Using the Particle Tracker, detected particles were compared to each other and the most likely particle track calculated for each detection.^(^^37^^)^ Detected particle tracks were then filtered to remove exceptionally short tracks (< 10 frames) deemed unlikely to provide useful data, and verified by eye to remove any tracks which were not bound within the confines of the cell, resulting in between 650 and1500 tracks per process/sheet.^(^^40^^)^ The precision of this tracking procedure was calculated from a series of 20 frames capturing stationary fluorescent particles. Tracking errors for *X* and *Y* coordinates were 23 (± 27) nm and 16 (± 17) nm, respectively for the TIRF imaging mode, and 21 (± 16) nm and 19 (± 16) nm, respectively, for images acquired in HILO mode. Thus, tracking errors were comparable between the TIRF and HILO configurations.

**Table 1.** ICY software tracking parameters used within this study

Parameter	Setting
Expected number of false positives (tracks)	30
Probability of detection	0.9
Expected number of new tracks per frame (tracks)	20
Expected number of new objects per frame (tracks)	50
Expected track length (frames)	20
Average particle displacement (pixels)	5
Minimum probability of existence	0.5
Threshold of termination	0.0001
Gate factor	4
Depth of track trees	4
Number of new tracks per frame (tracks)	15

*Note:* The parameters shown here were optimized on the oli-neu OL cell line and were utilized for all cells used within this study.^(^^31^^)^

Data from tracks were extracted in ICY bioanalysis using the Motion Profiler, Mean Squared Displacement, and Export to XLS function plugins. These operations provided the mean squared displacements (MSD) for each track (the average squared movement of a particle between two points), total displacement (summed movement over the entire track), the net displacement (straight line movement between the start and end points of the track), the track lengths, and the raw track data.^(^^36^^,^^38^^)^ The diffusion coefficient was calculated from MSD values using (1):(1)

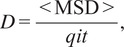

where *D* is the diffusion rate, and MSD is divided by the time (*t*) multiplied by the dimensionality constant (*q_i_*). In this case, *q_i_* is defined as “4” following the example of Ruthardt et al.^(^^41^^)^ for 2-dimensional imaging. We note that a single value of D was calculated for each track by averaging the values of *D* obtained from all available time intervals in the track. Although this approach provides a means to control bias caused by variability in the length of the captured tracks, we acknowledge it leaves open the possibility that *D* is over or underestimated for particles whose trajectories deviate from the linear MSD/*q_i_t* relationship expected by the equation, as would be the case for particles with restricted or enhanced diffusion. In this context, MSD versus time plots appear to show superdiffusive particle tracks within OPC processes and eOL bubbles, while the diffusion of particle tracks in mOL sheets appear relatively restricted (Supplementary Figure 1). These apparent deviations from normal diffusion may have introduced a degree of imprecision to our calculations of the diffusion coefficient. These limitations, and are our rational for selecting this equation, are considered in more detail in Section 4.

Equation (1) also assumes that particle tracking occurs in the absence of localization errors. As noted above, the localization methods used in this study are associated with a certain degree of error which will influence the diffusion coefficients. However, these localization errors are similar for both TIRF and HILO imaging modes, thus any imprecision in our calculations will have been equally distributed across the different cell types.

The confinement ratio was calculated using the track length and displacement values using (2):(2)

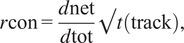

where net displacement *d*_net_ is divided by the total displacement *d*_tot_, then multiplied by track duration *t*_(track)_ to correct for the influence of track length as discussed by Beltman et al.^(^^42^^)^

The circularity ratio for each motion profile was calculated by tracing its outline and using the resulting polygon to determine values of area and perimeter. The circularity ratio was then calculated with (3):(3)

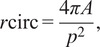

where four multiplied by pi and the area (*A*) is divided by the perimeter (*p*) squared to yield a circularity ratio (*r*_circ_) with a value between 0 and 1, with 0 being linear and 1 representing perfect circularity. A single motion profile was constructed for each imaged cell based on the motion of all of the particle tracks, hence circularity was assessed at the level of individual cells rather than the individual particle.

To aid the qualitative interpretation of these data, confinement ratios and diffusion coefficients were visualized as frequency distributions (% of particle population), with bins further categorized into color-coded groups. Confinement ratios naturally fell between 0 and 1, hence a set of 10 evenly sized frequency bins were selected. To determine the range of particle diffusion coefficients, all diffusion coefficient values from all conditions were combined to yield the range (9.34E-8 to 7.00E-2 μm^2^/s). These values were then divided between 12 equally spaced frequency bins. Confinement ratio frequency bins were categorized as being either light, medium, or highly confined. Similarly, diffusion coefficients were categorized into slow, intermediate, and fast diffusive particles, and the resulting groups are color coded in relevant histograms (Figures 3 and 5). These categorizations provide a simple aid to visualization and qualitative analysis of the data, but are not used on subsequent statistical analysis. The vast majority of MBP particles fell within the slowest diffusion bin (5.83E-04 μm^2^/s) (Supplementary Figure 2). Therefore, to aid visualization of faster diffusive particles, frequency distributions were graphed from the population falling within the remaining 11 bins. Nevertheless, statistical analysis for diffusion coefficients was computed on the entire population of particles.

### 2.7. Statistical analysis

Analysis focussed on comparing particle track circularity, particle confinement, and particle diffusion between the three stages of OL development: OPC processes, eOL membrane bubbles, and mOL sheets. Individual particle tracks were treated as biological replicates for analyses examining the behavior of individual particles (confinement and diffusion). For exploration of circularity, and for cell averaged analysis of particle confinement and diffusion (as noted in the text), individual cells were treated as biological replicates. Particle tracks were obtained from a total of 39 OPC processes, 23 eOL bubbles, and 27 mOL membrane sheets. Variance in the number of cells imaged for each cell type reflect the relative difficulty of generating and identifying cells at each maturational stage. A total of 24,721 OPC, 6,590 eOL, and 65,012 mOL MBP particle tracks were analyzed to assess particle confinement. For the diffusion analysis, a total of 32,320 OPC, 18,954 eOL, and 51,032 mOL particle tracks were analyzed. All statistical tests were computed with Prism 9.0 (Graphpad Software, El Camino Real, CA). Kolmogorov–Smirnov normality tests revealed that none of the data sets (particle track circularity, particle confinement, and particle diffusion, at all developmental stages) contained normal distributions. Therefore, nonparametric tests were used throughout the study and sample averages are reported as medians plus interquartile range (IQR, first to third quartiles). Multiple group comparisons (circularity data, confinement data) were investigated by the Kruskal–Wallis (KW) tests, and between-group differences checked by Dunn’s multiple comparisons test. For these tests, an alpha level of <0.05 was used for the detection of reliable differences. Frequency distributions for confinement and diffusion data were compared between pairs of groups by the Kolmogorov–Smirnov (KS) test for goodness of fit (e.g., OPC particle confinement data comparisons: OPC processes versus eOL bubbles; OPC processes versus mOL sheets). To correct for these multiple-group comparisons Bonferonni corrections were applied to alpha values as follows: original alpha value divided by the number of tests carried out on each sample (= 2), for example, original alpha *p* < 0.05 corrected to *p* < 0.025. Prism 9.0 software produced approximate *p* values for these KS tests, for example, *p* < 0.0001. Approximate *p* values were therefore referenced against the closest relevant Bonferroni corrected *p* alpha level, for example, observed *p* < 0.0001 referenced against *p* alpha 0.001 (corrected to *p* < 0.0005). Table 2 summarizes descriptive statistics (medians, IQR, 95% confidence limits for medians), and Table 3 summarizes statistical hypothesis tests for the data sets presented in the study.

**Table 2.** Summary of descriptive statistics aggregated from individual cells

ID	Measure	Structure	Median	95% CL of median	
A	Circularity	Not normal	OPC: 0.831	OPC: 0.776–0.879
			eOL: 0.599	eOL: 0.446–0.673
			mOL: 0.465	mOL: 0.446–0.480
B	Confinement ratio	Not normal	OPC: 0.464	OPC: 0.432–0.507
			eOL: 0.622	eOL: 0.525–0.726
			mOL: 0.257	mOL: 0.231–0.318
C	Diffusion	Not normal	OPC: 1.18 × 10^−4^	OPC: 1.04 × 10^−4^ – 1.44 × 10^−4^
			eOL: 9.02 × 10^−5^	eOL: 6.14 × 10^−5^ – 1.16 × 10^−4^
			mOL: 9.11 × 10^−6^	mOL: 7.65 × 10^−6^ – 1.35 × 10^−5^

*Note:* ID codes link table entries to in-text statistical results. Diffusion coefficient medians are expressed in μm^2^/s

**Table 3.** Summary of statistical tests

ID	Measure	Structure	Test	Test statistic/DF	*p*-value
A	Circularity	Not normal	KW	KW = 53.4	*p* < 0.0001
				DF = 2	
B	Confinement (particle distributions)	Not normal	KS		
			OPC vs. eOL	*D* = 0.074	*p* < 0.001^BF^
			OPC vs. mOL	*D* = 0.234	*p* < 0.001^BF^
			eOL vs. mOL	*D* = 0.290	*p* < 0.001^BF^
C	Confinement (cell medians)	Not normal	KW	KW = 29.51	*p* < 0.0001
				DF = 2	
D	Diffusion (particle distributions)	Not normal	KS		
			OPC vs. eOL	*D* = 0.034	*p* < 0.001^BF^
			OPC vs. mOL	*D* = 0.618	*p* < 0.001^BF^
			eOL vs. mOL	*D* = 0.597	*p* < 0.001^BF^
E	Diffusion (cell medians)	Not normal	KW	KW = 48.57	*p* < 0.0001
				DF = 2	

*Note:* ID codes link table entries to in-text statistical results. Diffusion coefficient medians are expressed in μm^2^/s.

Abbreviations: BF, Bonferroni correction for two comparisons; CL, confidence limits; D, Kolmogorov Smirnov goodness of fit test statistic; DF, degrees of freedom; KS, Kolmogorov Smirnov goodness of fit test; KW, Kruskal Wallace test statistic.

## 3. Results

Dendra2-tagged MBP was imaged at three stages of OL maturation: OPC, early myelinating OL (eOL), and mature myelinating OL (mOL) (see Figure 1 and Methods for details). Imaging was focused on processes (OPC), membrane bubbles (eOL), and membrane sheets (mOL) (see Methods). Three particle tracking parameters were investigated to describe MBP behavior in these regions: (1) Directionality, being the extent to which a particular direction is maintained during particle movement; (2) Confinement, being the degree to which particle movement is localized to a start-point, with low values reflecting constrained movement; and (3) Diffusion coefficient, a measurement of how quickly the particle will be moving over the time it is being tracked.

### 3.1. Directionality

**Figure 2.**
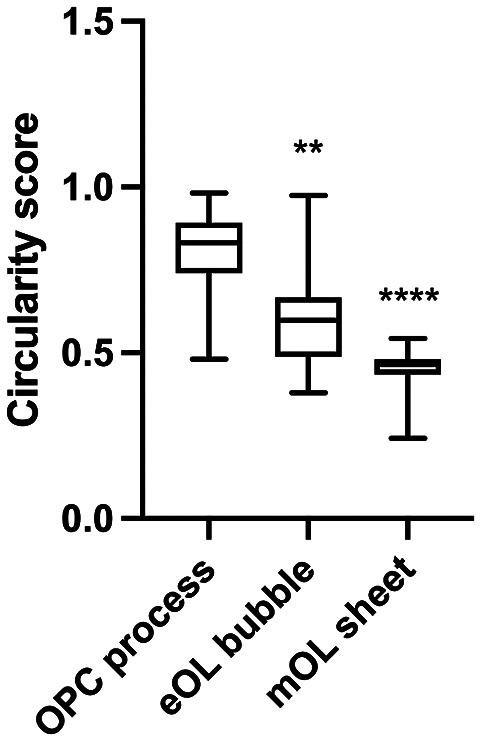
Box and Whisker plot depicting circularity scores derived from MBP motion profiles. Box ends show 25th and 75th percentile, middle bar shows medians, bar ends show minimum and maximum values. ** indicates post hoc comparison between eOL and OPC at p < 0.001, **** indicates post hoc comparison between OPC and mOL at p < 0.0001.

One of the key aspects of MBP behavior is directionality, which can indicate information on the protein interactions, messaging, and transport systems influencing the particle behaviors. MBP tracks within OPC processes show little to no directionality. This is summarized in the motion profile where the displacement of the particle track from its start position is plotted on an *X* and *Y*-axis, where the XY intercept is the starting position of the particle (Figure 1b-i), and on a vector diagram (Figure 1c-i) where tracks are shown to move almost equally within all directions, signifying random isotropic motion. This observation is fortified by circularity measurements of OPC MBP tracks, which show that the distribution of tracks is highly circular with a median value of 0.83 (IQR 0.78–0.88) (Figure 2 and Table 2,A). At the eOL stage, once myelination starts and cell processes begin to flatten into membrane bubbles, MBP particles begin to show a modest level of directionality (Figure 1b-ii,c-ii), that is accompanied by a change in circularity, whose median value falls to 0.60 (IQR 0.45–0.68) (Figure 2 and Table 2,A). The increase in directional movement continues with the transition from eOL to mOL with the myelin sheets of mOL displaying the most linear particle motion plots (Figure 1b-iii,c-iii), and the lowest median circularity scores (0.47, IQR 0.45–0.48) (Figure 2 and Table 2,A). Visual inspection of median circularity scores revealed a progressive maturation-dependent reduction, a view supported by statistical analysis revealing significant variance between the median circularity scores for OPC, eOL, and mOL (*p* < 0.0001, Table 3,A and Figure 2). Moreover, post hoc testing revealed that circularity values from eOL and mOL differed from OPC (*p* < 0.01 and *p* < 0.00001, respectively). However, the apparent decrease in circularity scores between eOL and mOL did not reach significance (*p* = 0.07). Overall, MBP particle directionality appears to transition from circular (random) motion to linear (directed) motion at the same time that cell morphology changes from a linear bipolar morphology (OPC) to a circular flattened sheet (mOL).

### 3.2. Confinement

**Figure 3.**
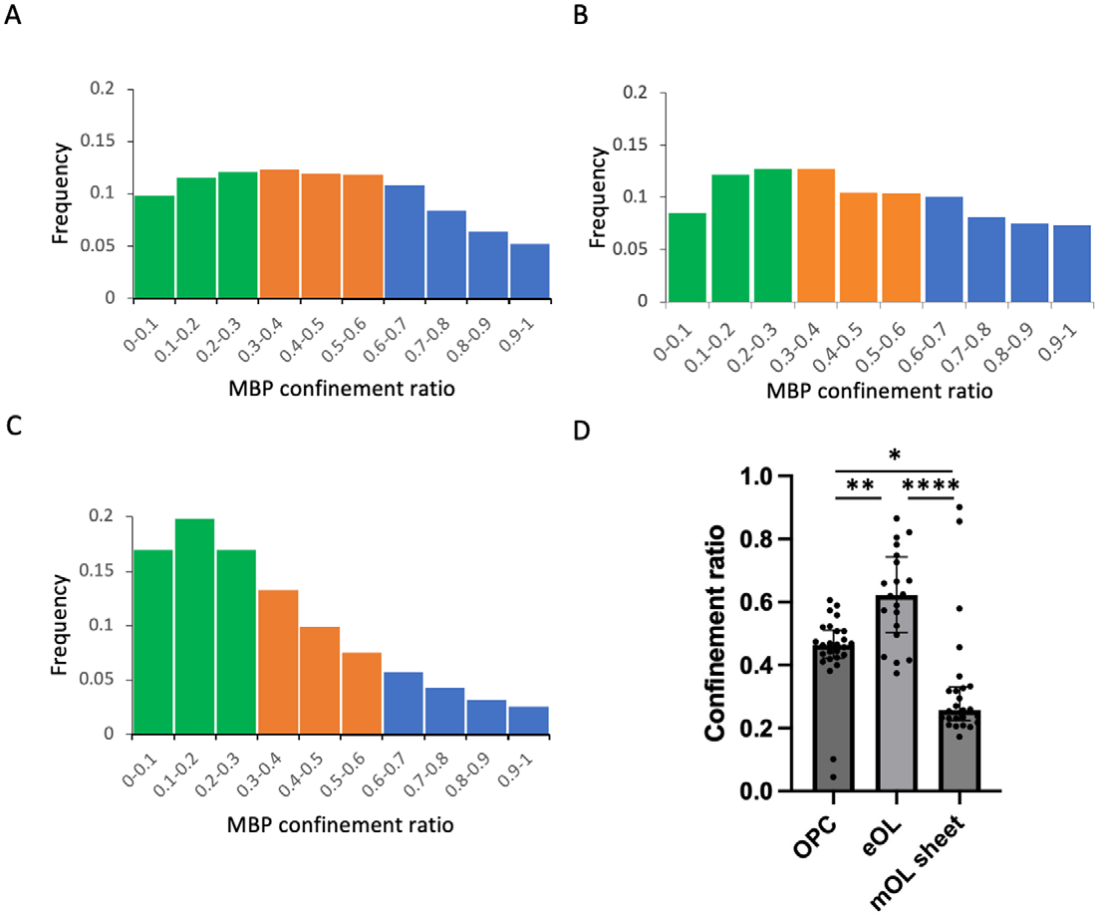
Developmental change in MBP particle confinement. Confinement histograms of MBP particles derived from OPC processes (a), eOL membrane bubbles (b), and mOL membrane sheets (c). Particle confinement is divided into three groups based on confinement (Methods): highly confined (green), moderately confined (orange), and lightly confined (blue). (d) Confinement ratios for OPC processes (OPC), eOL bubbles (eOL), and mOL sheets expressed as median values per cell. Individual data points show medians from individual cells. Bar shows median of the median for each cell type. Error bars show IQR. *, **, and **** indicates significance for Dunn’s multiple comparison tests at p < 0.05, p < 0.01, and p < 0.0001, respectively.

The direction of movement within the system is not the only aspect of MBP behavior which changes during maturation of OLs. Confinement, defined here as the extent to which movement is localized to a start-point, indicates whether particles are traveling away from their starting point, or simply moving about this location. Low confinement ratios indicate a high degree of confinement, while high ratios indicate particles with lower levels of confinement. Comparison of the frequency distributions for MBP confinement ratios reveals clear shifts at each developmental stage (Figure 3). Within OPC processes, the confinement behavior of MBP particles is distributed widely across a range of confinement ratios (Table 2,B), with the median value lying at 0.46 (IQR 0.42–0.51) (Figure 3a). As the cells mature to the eOL stage this distribution changes slightly with a modest shift toward particles with lower confinement (median ratio value rises to 0.62; IQR 0.50–0.74) (Figure 3b, high ratios/blue region). Confinement ratios show a more pronounced shift in the mOL stage with the distribution reversing to one containing more highly confined particle (median ratio falls to a value of 0.26; IQR 0.22–0.33) (Figure 3c, compare green vs. blue regions). Pair-wise comparison of these distributions by the KS test reveals significant differences at each transition (Table 3,B) indicating that these distributions arise from different populations of particles. This finding is also supported by an analysis of median confinement values by the KW test (Table 3,C and Figure 3d), which revealed that eOL exhibit significantly higher confinement ratios compared to OPC, indicative of a reduced level of confinement in eOL membrane bubbles versus OPC processes (OPC vs. eOL *p* < 0.0001). Similarly, confinement values from mOL were significantly lower when compared to either eOL (*p* < 0.0001), and OPC (*p* < 0.0001), indicating that the greatest levels of confinement were observed in mOL membrane sheets. In agreement with this, MSD versus time plots suggest reduced diffusion of MBP particles imaged in mOL sheets in comparison to OPC processes and eOL bubbles (Supplementary Figure 1). In conclusion, these results show that MBP confinement values are polarized between the eOL and mOL stages with the lowest degree of confinement found in eOL bubbles (highest ratios), and the greatest degree seen in mOL sheets (lowest ratios).

### 3.3. Diffusion

**Figure 4.**
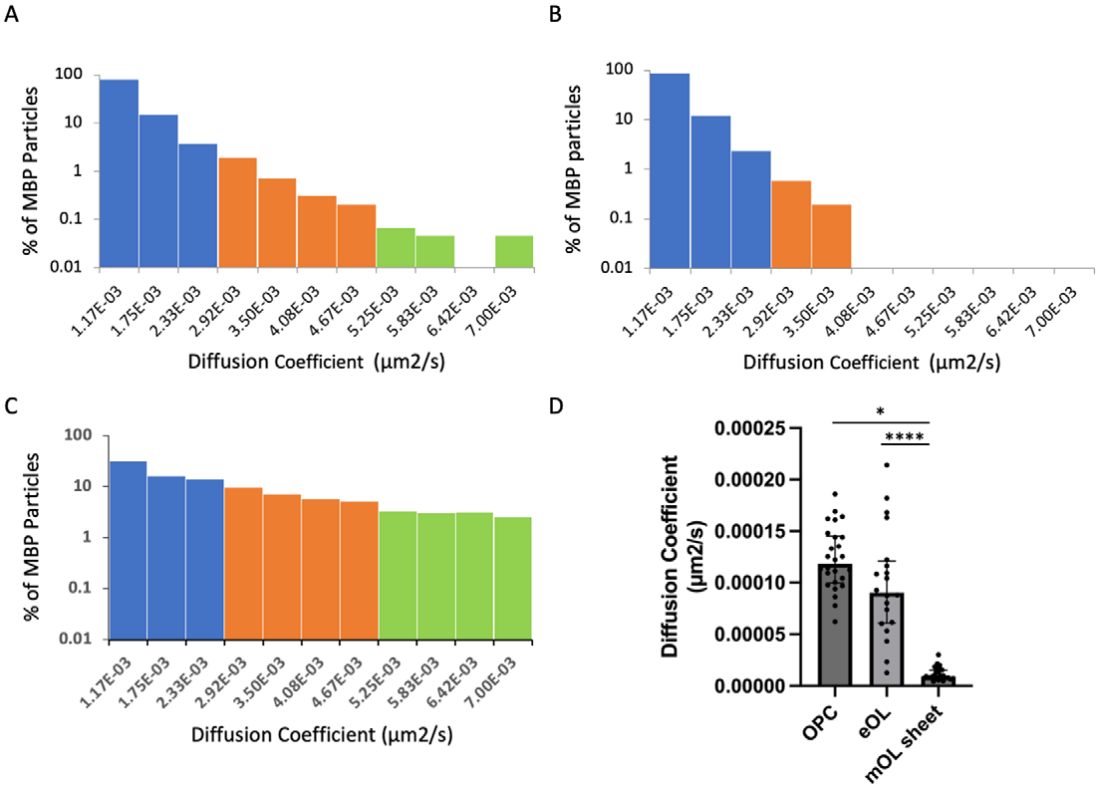
Developmental change in MBP particle diffusion. (a–c) Diffusion histograms of MBP particles derived from OPC processes (a), eOL membrane bubbles (b), and mOL membrane sheets (c). Particle diffusion is divided into three groups based on mobility: slow-moving (blue), intermediate-moving (orange), and fast-moving (green). Y-axis values (% MBP particles) are plotted on a log scale to allow clear visualization across the range of diffusion coefficients (X-axis). Note, data from the slowest particle bin (5.83E-04) are excluded from graphs but not from statistical analysis (Methods). (d) Diffusion coefficients for OPC processes (OPC), eOL bubbles (eOL), and mOL sheets expressed as median values per cell. Individual data points show medians from individual cells. Bar shows median of the median for each cell type. Error bars show IQR. * and **** indicates significance for Dunn’s multiple comparison tests at p < 0.05 and p < 0.0001, respectively.

**Figure 5.**
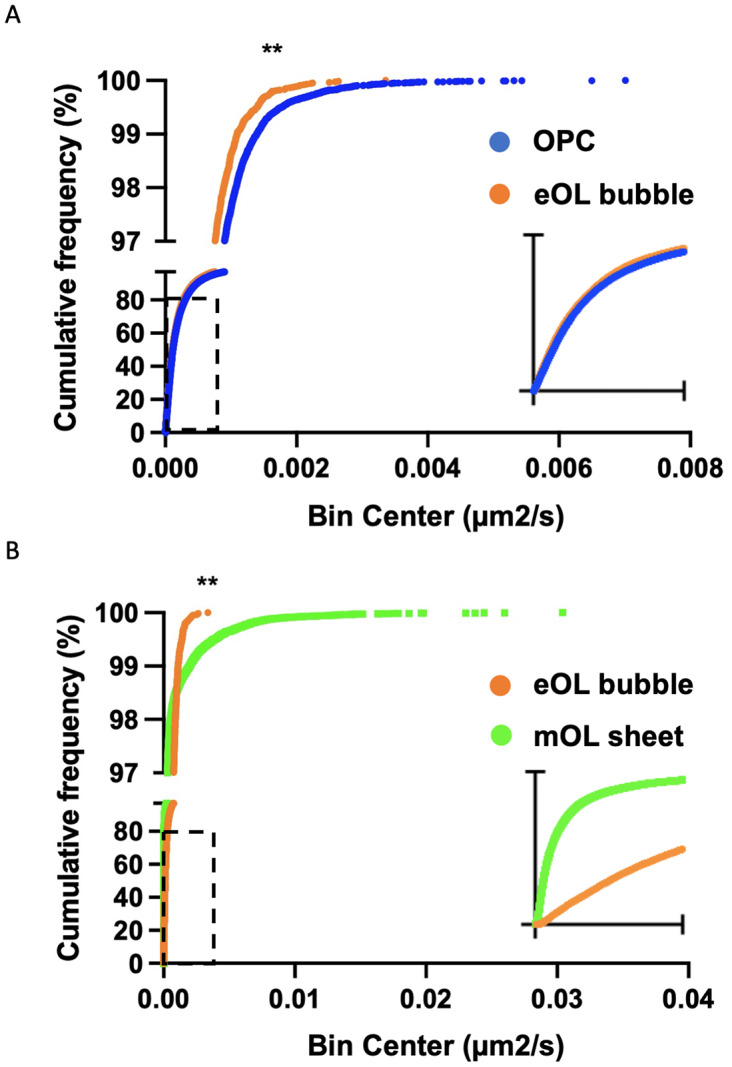
Cumulative frequency plots comparing diffusion coefficient distributions for particles imaged in OPC and eOL bubbles (a), and eOL bubbles versus mOL sheets (b). Insets in (a) and (b) show dashed regions on a smaller X-axis scale, range 0 to 4 × 10^−4^ and 0 to 1 × 10^−4^ μm^2^/s, respectively. ** indicates significance for KS comparison at p < 0.001.

While a particle may show a high degree of confinement this does not imply the absence of motion. Indeed, confined particles can exhibit a large amount of movement localized around their start position. In this context, diffusion coefficients provide a useful means to gauge the external forces that shape the motion of the particles under study. Of note, the diffusion coefficient reports the rate of movement rather than the fraction of particles moving. The diffusion rate of MBP particles in OPC processes show a broad range of diffusion constants ranging from slow-moving particles (Figure 4a, blue) which dominate the profile, to smaller subpopulations of particles moving at intermediate (Figure 4a, orange), and faster speeds (Figure 4a, green). Following differentiation, slow-moving particles still constitute the majority of particle imaged in eOL membrane bubbles, however, the smaller subpopulations of particles with intermediate speeds are reduced in abundance, while those with the highest diffusion are lost entirely (Figure 4b, orange and green). The absence of these highly diffusive particles is clearly apparent in cumulative frequency plots comparing the distributions of the most diffusive OPC and eOL particles (Figure 5a), and in the results of a pair-wise KS comparison (Table 3,D) (*p* < 0.001). Further differentiation leads to additional changes with a clear return in the intermediate and fast diffusive MBP particles imaged in mOL membrane sheets (Figure 4c). The return of the most highly diffusive particle is clearly visible in cumulative frequency plots (Figure 5b), and is reflected in the results of a pair-wise KS comparison (Table 3,D) (*p* < 0.001). Interestingly, MSD versus time plots show relatively suppressed diffusion in mOL sheets compared to OPC processes and eOL bubbles, although highly diffusive particles are still readily observed in mOL (Supplementary Figure 1D). Moreover, when aggregated at the individual cell level, mOL membrane sheets displayed the lowest median diffusion values (Table 2,C). Indeed, mOL sheets display significantly lower median diffusion values compared to OPC processes (*p* < 0.05) and eOL bubbles (*p* < 0.0001) (Figure 4d and Table 3,E). Taken together, these data emphasize that the fastest diffusive particles that are enriched in mOL represent a small subpopulation whose presence would go undetected by bulk measurements.

## 4. Discussion

Using SMT methods we show that several parameters of MBP particle motion are altered during OL maturation. Of note, the movement of MBP particles is strongly related to the level of OL differentiation, with the bulk of MBP molecules displaying a higher degree of confinement, and a progressive increase in directed motion, as maturation proceeds. Coexisting with these trends was the emergence of a subpopulation of fast diffusive particles in mature OL membrane sheets, whose relevance may be explained in terms of current models of MBP function (discussed below).

We explored directionality by inspecting the motion profiles and circularity of MBP particle tracks. Directionality is an important measure of particle movement. As shown by actin molecules, directional motion provides an indication as to whether a given particle is subject to molecular interactions, be they attractive or repulsive,^(^^43^^–^^45^^)^ while nondirectionality suggests an inert state devoid of molecular interactions. MBP particles are shown to have little to no directionality in OPC processes suggesting that this internal environment offers few opportunities for MBP to engage in molecular interactions. This behavior changes following differentiation to the eOL stage, where MBP molecules in membrane bubbles display more linear motion profile plots indicative of directed motion. This observation suggests the emergence of forces or interactions within the intracellular environment that play a role in directing MBP motion. This effect intensifies as the cells develop flattened membrane sheets, the in vitro equivalent of the myelin sheath, wherein MBP molecules show the greatest degree of directionality. Of note, this directionality is biased toward the periphery of the cell, consistent with a role for MBP in driving the expansion and wrapping of the myelin sheath.^(^^8^^)^ Interestingly, a recent model of myelin compaction posits diffusion of MBP particles away from sites of mRNA translation, at the innermost region (adjacent to the axon), and toward the outer lying wraps where compaction initiates.^(^^2^^)^ Knowledge of the mechanisms that direct this diffusion remain unknown, but it seems unlikely to involve cytoskeletal elements given the absence of tubulin and actin structures in compacting myelin.^(^^8^^)^ Nevertheless, OL membrane sheets display a highly polarized structure of compact and noncompact myelin that mirrors the structure of myelin in vivo,^(^^7^^)^ and while the spatial details of compaction in this environment remain to be determined, it is reasonable to consider whether the directed MBP diffusion observed in this environment reflects recruitment to sites of ongoing compaction.

The confinement behaviors we derived from the MBP particle tracks further increases the information we have on the system. MBP molecules within OPC processes exhibit a broad range of confinement ratios, implying many MBP molecules that are moving freely, and experiencing few forces and interactions that may influence their diffusion, or direct their behavior. This view agrees with the motion profile and circularity data describing a population of MBP particles seemingly unperturbed by forces capable of causing directionality in their motion. When cells mature to the eOL stage the distribution of confinement ratios shifts to higher values, implying a population of MBP particles with greater mobility. These changes are reversed at the mOL stage where MBP particles in membrane sheets appear more confined, and where MSD plots suggest restricted diffusion. This decrease in mobility may reflect interactions with other myelin proteins whose expression emerges as OL mature and myelination commences. Candidate proteins for this interaction include proteolipid protein (PLP), 2,3-cyclic nucleotide 3-phosphodiesterase (CNPase), and MBP itself, all of which have been shown to interact with MBP.^(^^9^^,^^46^^,^^47^^)^

Our observations of MBP particle behavior are obtained from distinct membrane structures (processes, bubbles, sheets). These structures arise sequentially during OL development, with bubbles and sheets arising from expansions of the OPC plasma membrane. Considering this, one could argue that the changes in particle behavior reflect changes in morphology. For example, confinement reduces as the space available for particle movement expands from the OPC process to the eOL bubble. However, this pattern disappears at the next maturational transition, when the membrane bubble expands further into the membrane sheet. Also countering a morphological explanation, our observations of directionality indicate increased directed motion as the narrow volume afforded by the OPC process expands into the larger volumes of membrane bubbles and sheets. Similarly, MBP particle diffusion exhibits a biphasic response (slowing, then increasing) with progressive membrane expansions that seem difficult to explain solely on the basis of morphological changes.

The diffusion rates of MBP allow observation of the rate particles are moving at. Diffusion data are useful as they provide information on particle interactions within the system, and on the strength of forces acting on the particles. MBP diffusion data from OPC reveal that the majority of particles at this stage are slow-moving, with only a small number of particles exhibiting high diffusion rates. Interestingly, the faster-moving members of the system are lost following differentiation to the eOL phenotype. This is suggestive of elements within eOL membrane bubbles that hinder the movement of MBP molecules. These hindrances may stem from physical interactions with intracellular complexes, or molecular interactions with binding partners such as PIP2^(^^8^^)^ and other MBP molecules.^(^^45^^)^ In line with this idea, the Simons lab demonstrated that MBP self-associates to create an intracellular meshwork that drives myelin compaction.^(^^6^^)^These self-interactions may explain the loss of faster particles in eOL membrane bubbles, and the developmental increase in confinement that reaches a peak in mature membrane sheets, an environment where myelin compaction is expected to be greatest. Interestingly, a population of the most diffusive particles, that are absent in eOL membrane bubbles, return in mature membrane sheets. The presence of highly diffusive particles may, on first consideration, appear at odds with the higher levels of confinement seen at this stage (e.g., Figure 3d). However, this discrepancy can be explained by the fact that the fast-moving particles represent a minor subpopulation of the whole, hence, their motion is unlikely to influence confinement values. Importantly, this subpopulation disappears when particle diffusions are aggregated at the level of individual cells, highlighting the ability of the single particle tracking approach to reveal complex behaviors that are obscured in ensemble-averaged analyses.

The reappearance of faster-moving particles suggests differences exist between the molecular environments offered by eOL membrane bubbles and mature membrane sheets. Considering their smaller size, membrane bubbles would require relatively smaller amounts of movement for MBP particles to be incorporated into the MBP matrices described by Aggarwal et al.^(^^6^^)^ In addition, live-imaging of similar membrane bubbles in myelinating cocultures^(^^32^^)^ indicate that these bubbles represent an early stage of myelination preceding the dramatic extension of membrane that characterizes myelin wrapping and lateral expansion. Thus, in our cultures, the expansion of the mature OL membrane sheet may involve, and in turn stimulate, the diffusion of a subpopulation of faster-moving MBP particles toward the periphery where they engage with membrane-bound partners such as PIP2.^(^^8^^)^ Importantly, this interpretation is also consistent with the greater degree of directionality observed for MBP particles tracked in mature membrane sheets.

Our data from mature membrane sheets indicate that fast diffusive particles co-exist alongside slower highly confined MBP molecules. These diverse particle behaviors support a multifunctional view of MBP, where subpopulations of molecules engage in distinct activities simultaneously within the same structure, for example, self-assembly into the compaction-driving MBP meshwork,^(^^7^^)^ stimulation of membrane expansion via migration toward and binding with membrane-bound PIP2,^(^^8^^)^ and other signaling activities unrelated to myelination.^(^^9^^,^^10^^)^ Future studies combining MBP particle tracking with molecular and pharmacological manipulations of targets such as PIP2, will be required to translate these observational data into mechanistic insights.

In this study, we explored diffusion by averaging diffusion values across the time-course of particle tracks. While this approach afforded a clear view of the generalized behavior of individual particles, it did not exploit the full spectrum of information contained within the time series. A detailed view of discrete diffusion behaviors across the particle track would allow investigation of features such as anomalous diffusion, and various related models, that allow inferences to be drawn on the forces acting on macromolecules within cells.^(^^17^^,^^48^^)^ Although the present data set could support preliminary explorations on this topic, additional control groups and experimental features not employed in the present study (e.g., unconjugated Dendra2, alternative conjugates of MBP involving probes, such as quantum dots with longer photobleaching lifetime), are required for a more robust analysis. Future studies encompassing these requirements have the potential to expand our initial observations and yield further insight into the mechanisms governing MBP function within myelin.

To our knowledge, this is the first single particle tracking study to examine a myelin protein. Given the absence of information on particle behaviors from related tracking studies, we selected a diffusion equation that made few assumptions about the particles under study. Nevertheless, (1) assumes that the particles under study exhibit normal Brownian diffusion. Our MSD versus time plots (Supplementary Figure 1) suggest that a number of particles, particularly those in OPC processes and eOL bubbles, exhibit profiles that likely deviate from normal diffusion.^(^^49^^)^ Considering this, the diffusion coefficients calculated in this article may be imprecise. Nevertheless, we consider that (1) is a rational choice for a first study of MBP particle diffusion, and that the resulting diffusion coefficients provide a reasonable basis for the comparison of MBP particle behaviors across different stages of the OL lineage.

SMT methods have been employed to study a range of neurobiological processes in neurons including neurotransmitter vesicle release and recapture, actin dynamics within dendritic spines, and trafficking of glutamate receptors between synaptic and extra-synaptic sites.^(^^50^^)^ SMT has also been implemented in astrocytes. Here, particle tracking has helped to resolve the dynamic behavior of membrane proteins, including aquaporins, glutamate transporters, and metabotropic glutamate receptors whose localization in astroglial membranes plays key roles in regulating CNS function.^(^^51^^–^^53^^)^ In contrast to neurons and astrocytes, single particle tracking has not been employed in oligodendroglia, or the myelin structures they elaborate. Therefore, the workflow we describe opens new opportunities to expand our understanding of biological processes within cell types and membrane structures whose activities are essential to CNS function.

## 5. Conclusion

We have described a novel imaging approach involving SFV-mediated expression of Dendra2-tagged MBP, and fast acquisition TIRF and HILO imaging, that together allow tracking of single MBP particles in OPC processes, and OL membrane bubbles and membrane sheets. Using these methods, we revealed changes in the molecular dynamics of MBP during OL maturation. Quantification of single particle motion profile plots, MSD time plots, and particle confinement and diffusion data, show that MBP molecules alter their behavior showing greater confinement and complex changes in diffusion as OL develop membrane structures associated with myelination. However, mature OL membrane sheets also contain subpopulations of particles whose fast-diffusive behaviors are consistent with particle recruitment to proposed sites of myelin expansion and compaction. Future studies should refine this approach by exploring alternative modes of diffusion within populations of MBP particles, and expand on the observational results by identifying the molecular interactions that drive MBP particle confinement and diffusion during myelination. This mechanistic work holds great potential to improve fundamental knowledge of myelination, and uncover novel targets whose modulation may promote myelin formation.

## Supporting information

Rassul et al. supplementary material 1 Rassul et al. supplementary material

Rassul et al. supplementary material 2 Rassul et al. supplementary material

Rassul et al. supplementary material 3 Rassul et al. supplementary material

## Data Availability

All data files for time-lapse imaging and SMT analysis are publicly available from the BioImage Archive (BioStudies accession number S-BIAD914).

## Supplementary material

The supplementary material for this article can be found at https://doi.org/10.1017/S2633903X23000259.
