# Supplementary material for: Single-molecule tracking of myelin basic protein during oligodendrocyte differentiation
Source: Biol Imaging. 2023 Nov 20;3:e24. doi: 10.1017/S2633903X23000259 (PMC10951920; doi:10.1017/S2633903X23000259)
Supplement: Rassul et al. supplementary material 1 — Rassul et al. supplementary material [file S2633903X23000259sup001.docx]

Rassul et al. Supplementary Figures

**
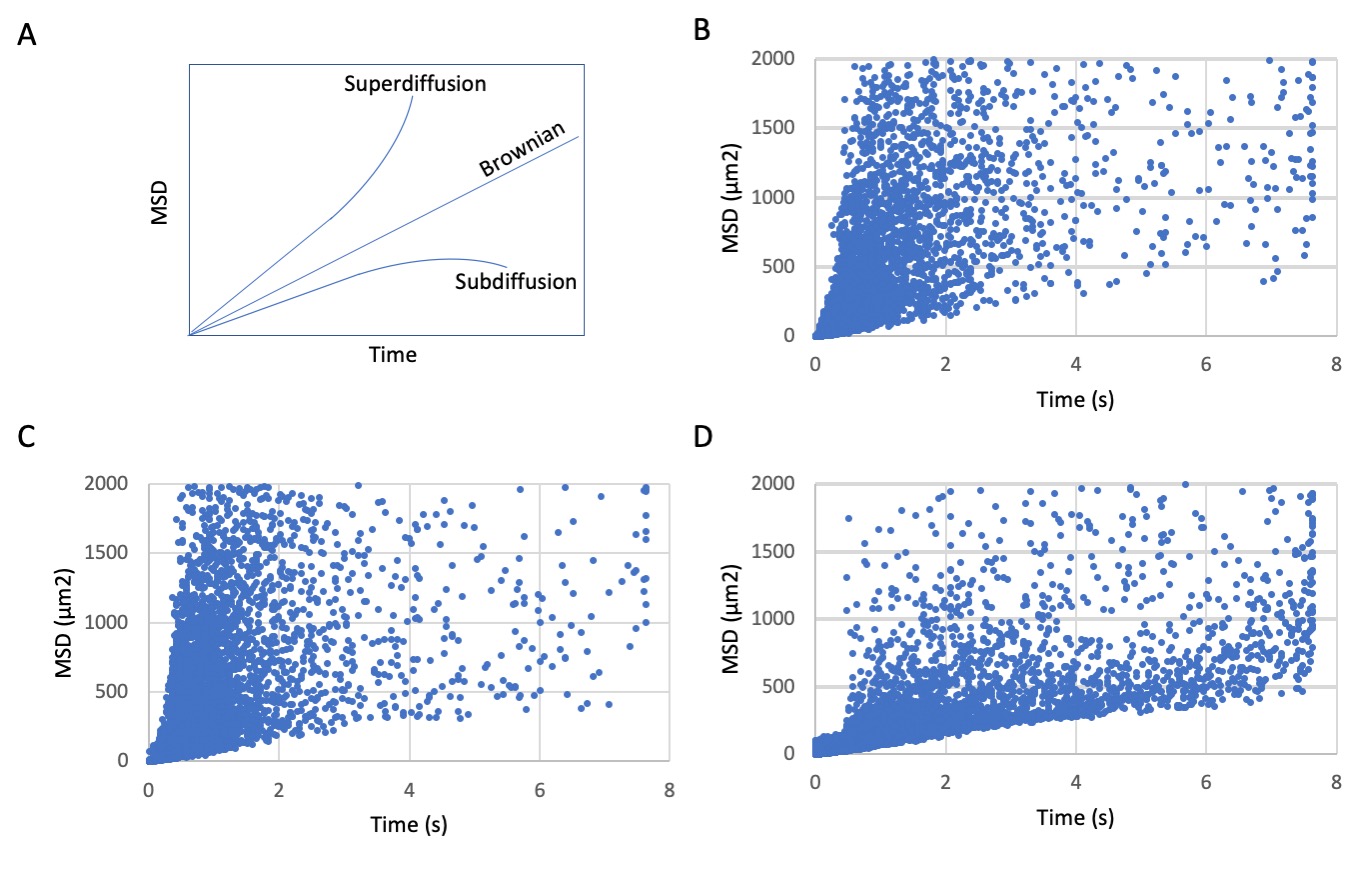
**

**Supplementary Figure 1.** MSD vs time plots reveal a shift in the diffusive behaviour of MBP particles during oligodendrocyte differentiation. A. Schematic showing possible modes of particle diffusion (adapted from ^49^). Particle tracks in OPC processes (B) and eOL bubbles (C) exhibit a tendency towards higher diffusivity, particularly over a shorter time span. The distribution of more highly diffusive particles is reminiscent of superdiffusion. D. Particle tracks imaged from mOL sheets exhibit a restricted form of diffusion relative to OPC processes and eOL bubbles. Graphs in B-D were generated from imaging fields taken from 5 to 6 individual cells per cell type. Individual data points indicate unique particle tracks.


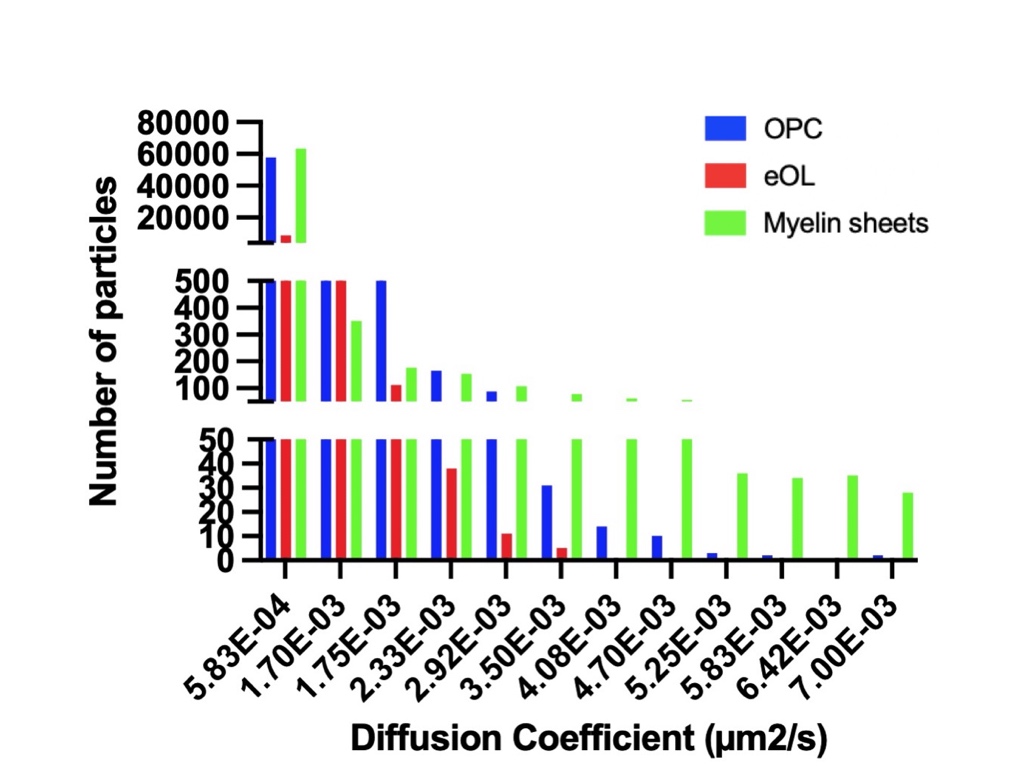


**Supplementary Figure 2.** Distributions of MBP particle diffusion coefficients expressed as raw particle numbers for OPC processes, eOL membrane bubbles and mOL myelin sheets. Y axis is split to accommodate the range of particle numbers allocated across the distribution bins. Note, the vast majority of particles for all cell types fall within the slowest bin.
