# Supplementary figures and images for: Single-molecule tracking of myelin basic protein during oligodendrocyte differentiation
Source: Biol Imaging. 2023 Nov 20;3:e24. doi: 10.1017/S2633903X23000259 (PMC10951920; doi:10.1017/S2633903X23000259)

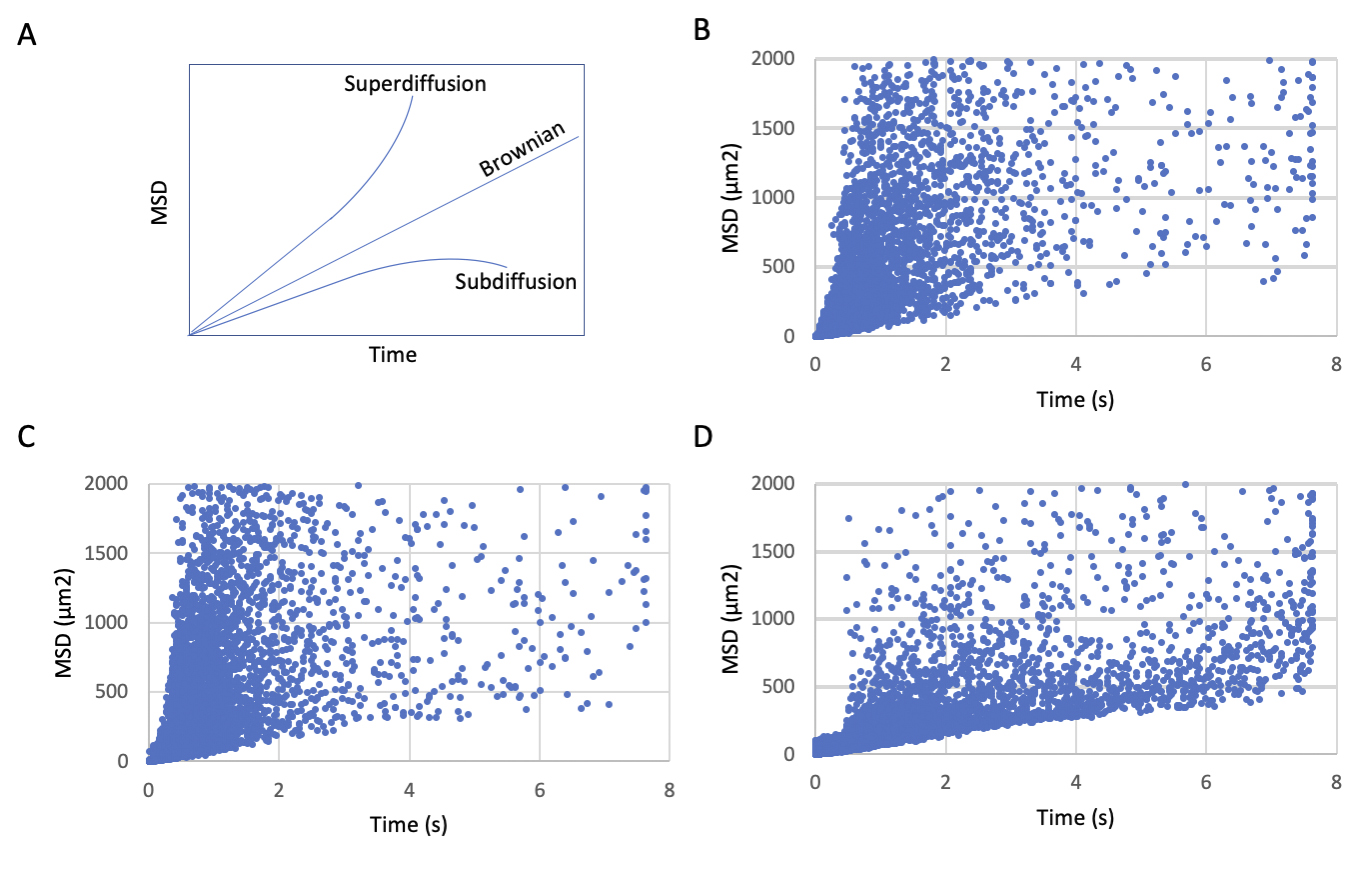

Supplement: Rassul et al. supplementary material 2 — Rassul et al. supplementary material [file S2633903X23000259sup002.jpg]

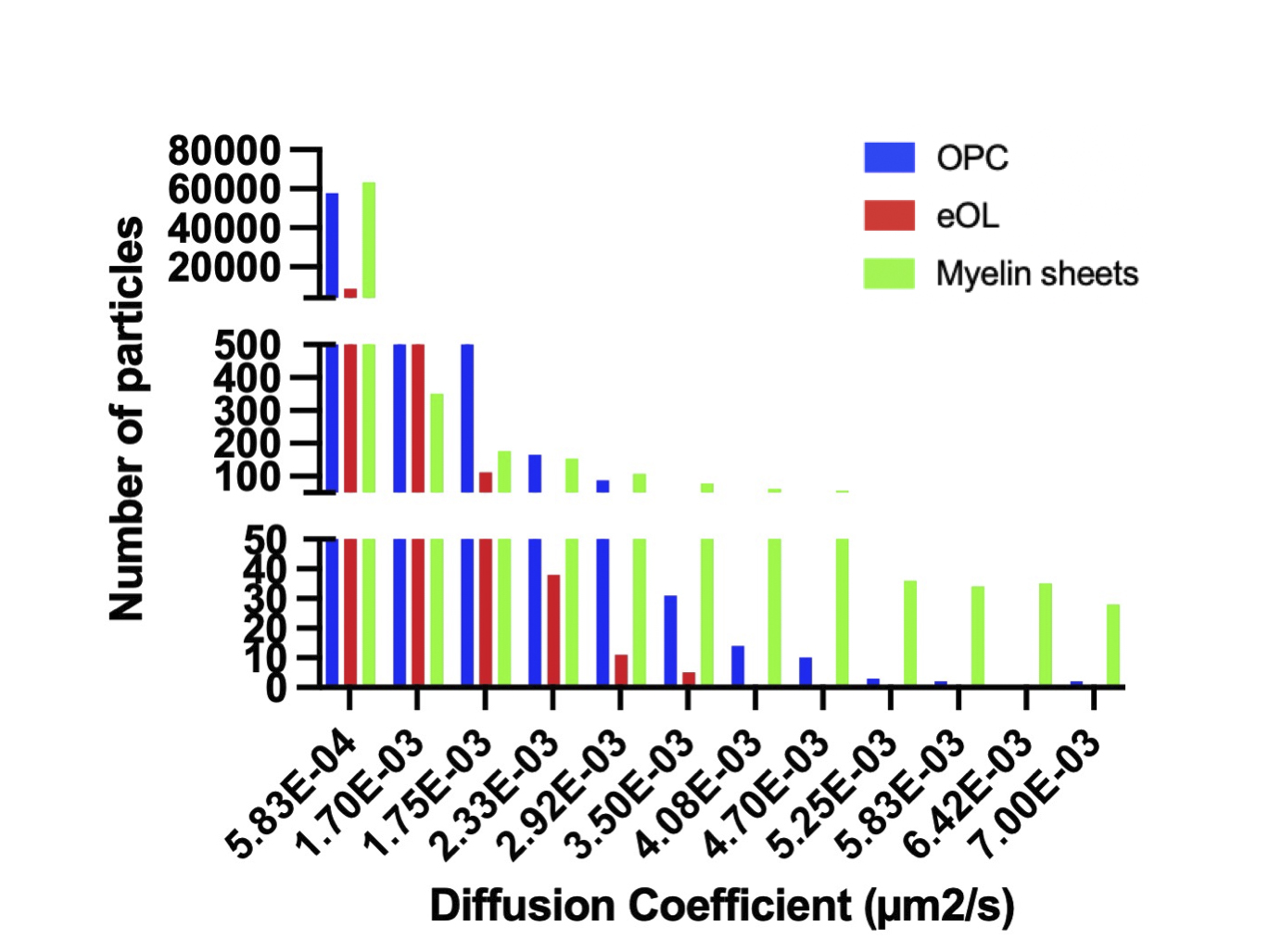

Supplement: Rassul et al. supplementary material 3 — Rassul et al. supplementary material [file S2633903X23000259sup003.jpg]
